# Metallo‐supramolecular nanofibers based on type‐I photosensitizer for synergistic antibacterial therapy

**DOI:** 10.1002/smo.20240037

**Published:** 2024-12-01

**Authors:** Shibo Lyu, Lukun Li, Jingdong Gao, Dapeng Liu, Fengling Song

**Affiliations:** ^1^ Institute of Frontier Chemistry School of Chemistry and Chemical Engineering Shandong University Qingdao China; ^2^ Department of Chemistry National University of Singapore Singapore Singapore; ^3^ School of Life Sciences Shandong University Qingdao Shandong China

**Keywords:** antibacterial therapy, metallo‐supramolecular, type‐I photodynamic therapy

## Abstract

Photodynamic therapy (PDT) has shown great merits in treating microbial infections due to its absence of bacterial resistance. However, the pronounced hypoxic microenvironment in the bacterial infections limits the therapeutic efficiency of traditional type‐II PDT, which is highly dependent on oxygen. Here type‐I photosensitizer BTZ_n_‐Py (*n* = 8, 20) coordinates with chemical antibacterial agent Ag^+^ to fabricate metallo‐supramolecular nanofibers. Under light irradiation, the formed nanofibers could not only generate type‐II reactive oxygen species (ROS), ^1^O_2_, but also produce type‐I ROS O_2_^•−^ which addressed the hypoxic issues within infected tissues. Moreover, the acid‐ and photo‐active Ag^+^ release from the nanofibers endowed the metallo‐supramolecular nanofibers with controlled release characteristic, which showed good biocompatibility to normal tissues. Owing to controlled Ag^+^ release and photoinduced type‐I ROS, the in vitro and in vivo experiments confirmed the significantly synergistic antibacterial performance of the metallo‐supramolecular fibers against both Gram‐positive and Gram‐negative bacteria.

## Graphical abstract

The metallo‐supramolecular nanofibers were self‐assembled from Type‐I photosensitizers BTZn‐Py (*n* = 8, 20) and chemical antibacterial agents Ag^+^ through coordination interactions to achieve synergistic antibacterial therapy.

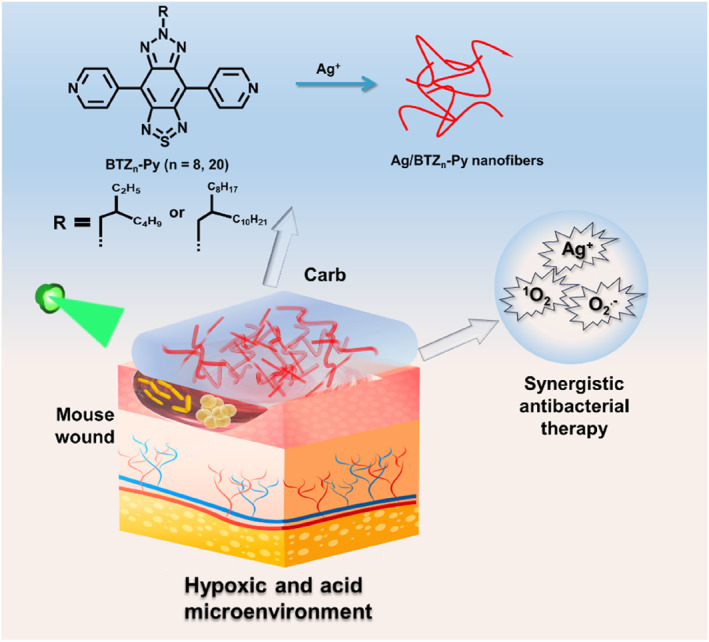

## 1 INTRODUCTION

Bacterial infections are a significant global public health concern.[^1^, ^2^, ^3^, ^4^] Antibiotic therapy is a key approach to treating bacterial infectious diseases.[^5^, ^6^, ^7^, ^8^] However, the overuse and misuse of antibiotics have led to the emergence of drug‐resistant bacteria, posing a serious threat to human health.[^8^, ^9^, ^10^] Recently, photodynamic therapy (PDT) has emerged as a promising antibacterial strategy due to its wide‐ranging antimicrobial effects, low toxicity, and high selectivity.[^3^, ^11^, ^12^] Importantly, it has also shown minimal development of bacterial resistance.

PDT is commonly categorized into type‐I PDT and type‐II PDT.[^13^, ^14^] In type‐I PDT, the photo‐induced photosensitizer can generate cytotoxic reactive oxygen species (ROS), such as superoxide anion radical O_2_^•−^ and hydroxyl radical •OH through electron transfer.^[15]^ While type‐II PDT can produce cytotoxic singlet oxygen (^1^O_2_) through energy transfer, which is highly oxygen‐dependent.^[2]^ However, the growth of bacteria and the immune responses of the host deplete nutrients and oxygen, leading to a hypoxic microenvironment in bacterial infections.^[16]^ This lack of oxygen in the infection sites hampers the efficacy of oxygen dependent type‐II PDT treatment.[^17^, ^18^] In contrast, type‐I PDT, which involves electron transfer, is less dependent on oxygen and presents significant advantages over type‐II PDT.

For most reported type‐I PDT photosensitizers,[^19^, ^20^] their short absorption wavelength and lifespan of the generated ROS have restricted their effectiveness in treating deep‐seated tissue infections.[^21^, ^22^] To improve the antibacterial efficacy of type‐I PDT photosensitizers, there is an urgent need to develop synergistic antibacterial strategies that combine PDT with other biochemical agents to combat bacterial infections effectively.

**SCHEME 1:**
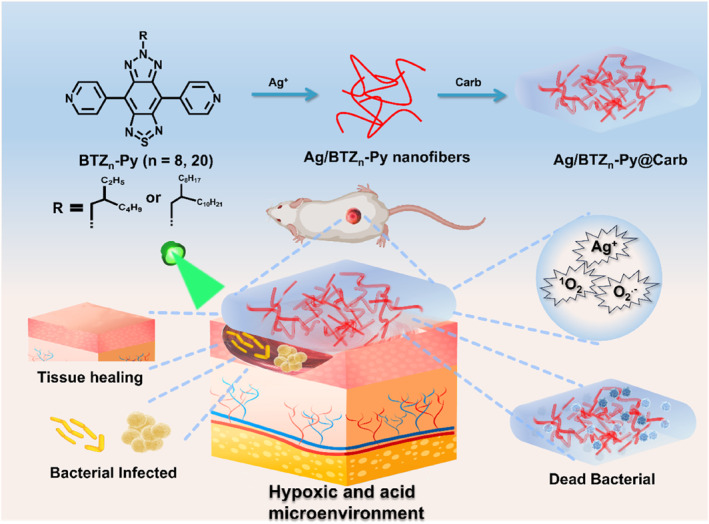
Schematic illustration of the formation of metal‐organic coordination nanofibers Ag/BTZ_n_‐Py (*n* = 8, 20) and their treatment of bacterial wound infection.

In this work, we designed and fabricated multifunctional Ag/BTZ_n_‐Py (*n* = 8, 20) nanofibers by combining the type‐I photosensitizers BTZ_n_‐Py (*n* = 8, 20) and the chemical agents Ag^+^. Under light irradiation, the formed nanofibers could not only efficiently produce type‐II ROS ^1^O_2_ but also type‐I ROS O_2_^•−^ which addressed the hypoxic issues in the bacterial infection microenvironments. Moreover, the nanofibers exhibited excellent biosafety towards noninfected tissues due to the controlled photo‐ and acid‐active Ag^+^ release. And the release amount of Ag^+^ was proved to be correlated with the lengths of alkyl chains in the photosensitizers BTZ_n_‐Py. Due to photoinduced type‐I ROS and controlled release Ag^+^, the Ag/BTZ_n_‐Py nanofibers mixing with the Carbopol 941 hydrogel exhibited outstanding synergistic antibacterial performance in eradication of Gram‐positive and Gram‐negative bacteria. The effective in vivo anti‐infective ability of the Ag/BTZ_n_‐Py (*n* = 8, 20)@Carb hydrogel was confirmed in a *S. aureus* and *E. coli*—infected wound model (Scheme 1).

## 2 RESULTS AND DISCUSSION

### 2.1 Synthesis and characterization of BTZ_n_‐Py (*n* = 8, 20) and nanofibers

**FIGURE 1:**
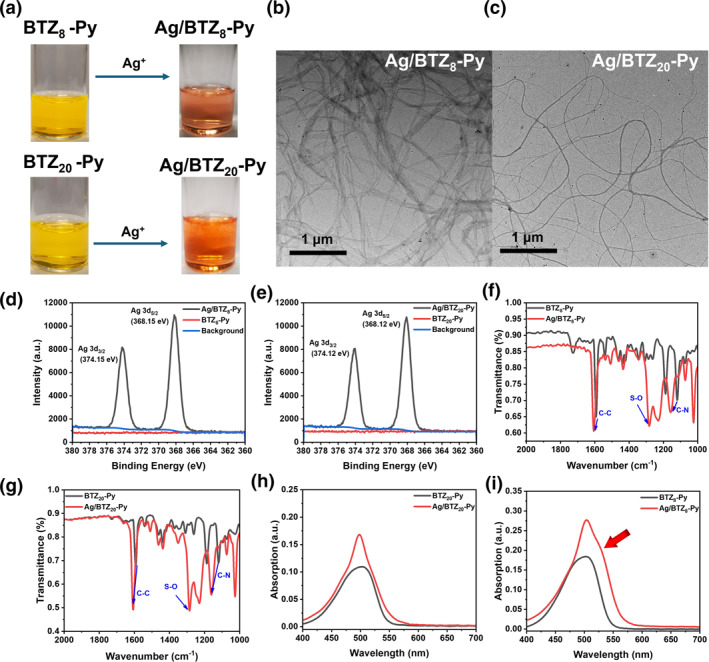
(a) Pictures of BTZ_n_‐Py before coordination and Ag/BTZ_n_‐Py after coordination in tetrahydrofuran (THF) solution. (b and c). TEM images of Ag/BTZ_8_‐Py and Ag/BTZ_20_‐Py. (d and e) The XPS spectra of Ag 3d_3/2_ and Ag 3d_5/2_ from BTZ_8_‐Py, BTZ_20_‐Py, Ag/BTZ_8_‐Py and Ag/BTZ_20_‐Py. (f and g) FTIR spectra of BTZ_8_‐Py, BTZ_20_‐Py, Ag/BTZ_8_‐Py and Ag/BTZ_20_‐Py. (h) Ultraviolet absorption spectra of BTZ_20_‐Py and Ag/BTZ_20_‐Py in THF solution. (i) Ultraviolet absorption spectra of BTZ_8_‐Py and Ag/BTZ_8_‐Py in THF solution.

The photosensitizer ligands BTZ_n_‐Py (*n* = 8, 20) with alkyl chains of different lengths were first synthesized according to the previous report.^[23]^ Details of the synthetic routes and structural characterizations including ^1^H NMR, ^13^C NMR, and mass spectrometry were provided in Figures S1–S3. Supramolecular aggregates Ag/BTZ_n_‐Py (*n* = 8, 20) were formed by simply addition of AgOTf (1 equiv of BTZ_n_‐Py) into the BTZ_n_‐Py solution in THF at room temperature (Figure 1a). Upon formation of Ag/BTZ_n_‐Py aggregates, the structurally characterized by Transmission Electron Microscope (TEM) and X‐ray Photoelectron Spectroscopy (XPS). TEM images (Figure 1b,c) of Ag/BTZ_n_‐Py aggregates revealed the formation of nanofibers with varying widths. Notably, the Ag/BTZ_8_‐Py nanofibers appeared to have a greater width compared to the Ag/BTZ_20_‐Py nanofibers. The higher magnification TEM images (Figure S4) confirmed that the Ag/BTZ_8_‐Py nanofibers were, in fact, bundles of fibers. Meanwhile, EDS analysis confirmed the uniform distribution of Ag, N and S in the formed nanofibers (Figure S5). XPS spectra (Figure 1d,e and Figure S6) further confirmed the elemental composition and chemical states of Ag/BTZ_n_‐Py nanofibers where the high‐resolution Ag 3d spectra of Ag/BTZ_n_‐Py display two major spin‐orbit doublets (3d_3/2_ at 374.2 eV and 3d_5/2_ at 368.2 eV). In the infrared spectra (IR), the peak (1120 cm^−1^) related to the C‐N stretching vibration in BTZ_n_‐Py shifted to 1155 cm^−1^ and 1160 cm^−1^, respectively, upon coordination with Ag^+^ (Figure 1f,g). Moreover, the IR spectra of Ag/BTZ_n_‐Py nanofibers showed a strong peak at 1285 cm^−1^ which was assignable to the S‐O stretching vibration in OTf^−^ (Figure 1f,g). These aforementioned structural characterizations confirmed that the self‐assembly of nanofibers were induced by the metal‐coordination between Ag^+^ and pyridine moieties in the BTZ_n_‐Py ligands. Interestingly, the addition of iodopropane to the formed nanofibers caused the solution color to revert to yellow, accompanied by the decomposition of the nanofibers (Figures S7 and S8). This observation suggests that the release of Ag^+^ ion would result in degradation of the nanowires, leading to the liberation of both BTZ_n_‐Py and Ag^+^.

The UV‐Vis spectra of the photosensitizer ligands BTZ_n_‐Py and their corresponding nanofibers were recorded in THF. As illustrated in Figure 1h,i, compared to the absorption peaks of the photosensitizer ligands, both absorption peaks of the self‐assembled nanofibers became sharper, accompanied by an increase in the molar absorption coefficient. Specifically, partial J‐aggregates were observed in the Ag/BTZ_8_‐Py nanofibers as evidenced by the appearance of the minor peak at 528 nm. The partial J‐aggregates indicated the order packings of the photosensitizer ligands BTZ_8_‐Py in the formed nanofibers. For Ag/BTZ_20_‐Py nanofibers, the amorphous packing of BTZ_20_‐Py can be attributed to the steric hindrance imposed by the elongated alkyl chains. Additionally, the nitrogen isothermal adsorption and desorption curves (Figure S9) revealed that the BET surface area of Ag/BTZ_20_‐Py nanofibers was 3.674 m^2^/g, significantly higher than that of Ag/BTZ_8_‐Py nanofibers 1.579 m^2^/g, indicating that there were more cavities in the Ag/BTZ_20_‐Py nanofibers. These findings indicated that partial J‐aggregates led to Ag/BTZ_8_‐Py nanofibers exhibiting a more densely packed molecular structure. Moreover, the pore sizes measured by Barret‐Joyner‐Halenda (BJH) method were 2.192 nm for Ag/BTZ_8_‐Py and 2.190 nm for Ag/BTZ_20_‐Py, respectively (Figure S9), which demonstrated that the formed metallo‐supramolecular fibers were mesoporous structures.

### 2.2 ROS generation and Ag^+^ release upon light irradiation

**FIGURE 2:**
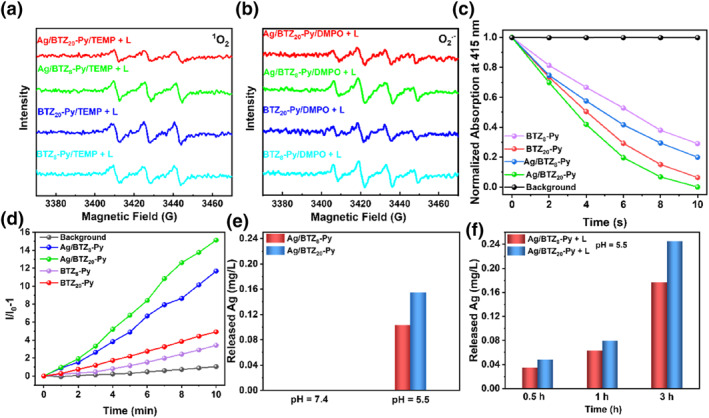
(a) electron spin resonance (ESR) spectrum of Ag/BTZ_8_‐Py and Ag/BTZ_20_‐Py in ethanol using TEMP as an ^1^O_2_ trap in the presence of 515–530 nm LED. (b) ESR spectrum of Ag/BTZ_8_‐Py and Ag/BTZ_20_‐Py in ethanol using DMPO as an O_2_^•−^ trap in the presence of LED (515–530 nm, 20 mW cm^−2^). (c) 515–530 nm LED time‐dependent degradation of DPBF mixed with BTZ_8_‐Py, BTZ_20_‐Py, Ag/BTZ_8_‐Py and Ag/BTZ_20_‐Py under LED (515–530 nm, 20 mW cm^−2^) within time‐dependent. (d) FL emission spectrum of DHR‐123 mixed with Ag/BTZ_8_‐Py and Ag/BTZ_20_‐Py under LED (515–530 nm, 20 mW cm^−2^) within time‐dependent. (e) Ag^+^ release profile from Ag/BTZ_8_‐Py and Ag/BTZ_20_‐Py in pH 7.4, 5.5 under 3 h (f) Ag^+^ release profile from Ag/BTZ_8_‐Py + L and Ag/BTZ_20_‐Py + L in pH 5.5 under different time (0.5 h, 1 h, 3 h).

Using electron spin resonance spectroscopy, the ROS type produced by BTZ_n_‐Py and Ag/BTZ_n_‐Py nanofibers was confirmed. Upon irradiation with an LED lamp (515–530 nm, 20 mW cm^−2^), Ag/BTZ_n_‐Py nanofibers exhibited distinctive triplet peaks which was ascribed to singlet oxygen (^1^O_2_) (Figure 2a and Figure S10). By using 2,2imethyl‐3,4ihydro‐2H‐pyrrole1‐oxide (DMPO) as a capture probe for O_2_^•**−**^ (Figure 2b and Figure S11), the characteristic signals were detected as well. This suggested that both type‐I and type‐II ROS was generated by Ag/BTZ_n_‐Py nanofibers under light irradiation. The cyclic voltammetry was employed to confirm type‐I PDT pathway to generate O_2_^•−^. The redox potential of the O_2_/O_2_^•−^ couple is known to be −0.33 V. As shown in Figure S12, Cyclic voltammetry measurements indicate that BTZ_8_‐Py (−0.61 V), BTZ_20_‐Py (−0.64 V), Ag/BTZ_8_‐Py (−0.61 V), and Ag/BTZ_20_‐Py (−0.63 V) possess suitable reduction potentials, allowing them to transfer electrons to O_2_, thereby generating O_2_^•−^.

To further investigate the degradation rate of ROS, 1,3‐ diphenylisobenzofuran (DPBF) was used as a singlet oxygen ^1^O_2_ probe. Upon exposure to LED irradiation, the characteristic absorption peak of DPBF at 415 nm gradually decreased after interaction with ^1^O_2_ generated by Ag/BTZ_n_‐Py (Figure 2c, Figure S13). Notably, Ag/BTZ_20_‐Py almost completely degraded DPBF within 10 s of illumination, whereas Ag/BTZ_8_‐Py required less than 20 s for complete degradation. Additionally, the production efficiency of O_2_^.‐^ was detected using the type‐I ROS probe dihydrorhodamine‐123 (DHR‐123). As shown in Figure 2d and Figure S14, after 10 min of LED illumination (515–530 nm, 20 mW cm^−2^), the fluorescence emission intensity of DHR‐123 mixed with Ag/BTZ_n_‐Py nanofibers significantly increased where Ag/BTZ_20_‐Py nanofibers exhibited stronger O_2_^•−^ production ability. Furthermore, compared to the ROS generation rate of the photosensitizer ligand BTZ_n_‐Py, the prepared Ag/BTZ_20_‐Py nanofibers exhibited a higher ROS generation efficiency.[^24^, ^25^] This could be attributed to the heavy atom effect of silver ions, which enhanced the spin‐orbital coupling effect.

Afterword, the release amount of Ag^+^ ions as a function of time at different conditions was plotted. The released concentration of Ag^+^ was tested by inductively coupled plasma‐optical emission spectroscopy (ICP‐OES). In the dark group (Figure 2e), an obvious increase in Ag^+^ concentration was observed in a pH 5.5 solution, similar to that of the microenvironment of bacteria, suggesting that the acidic environment facilitates the release of Ag^+^. And the negligible release amount was detected in pH 7.4 buffer solution, indicating the low toxicity of the nanofibers towards noninfected tissues. In terms of light irradiation group (Figure 2f), a progressive increase in Ag^+^ concentration was observed in the duration of irradiation compared to dark group. Moreover, the release rate of Ag/BTZ_20_‐Py was always larger than Ag/BTZ_8_‐Py, which could be attributed to more cavities in the formed Ag/BTZ_20_‐Py nanofibers. All the Ag^+^ releasing results confirmed the controlled Ag^+^ release characteristics in response to the acid microenvironment and light irradiation. Meanwhile, the ROS generation ability of the liberated BTZ_n_‐Py after Ag^+^ release was also tested. Upon addition of iodopropane into the formed nanofibers, the supernatant was collected and evaluated using DPBF and DHR 123. As shown in Figure S15, the supernatant consisting of the liberated BTZ_n_‐Py maintained excellent ROS (^1^O_2_ and O_2_^•−^) generation capacity.

### 2.3 In Vitro photodynamic antibacterial activity of Ag/BTZ_n_‐Py (*n* = 8, 20).

**FIGURE 3:**
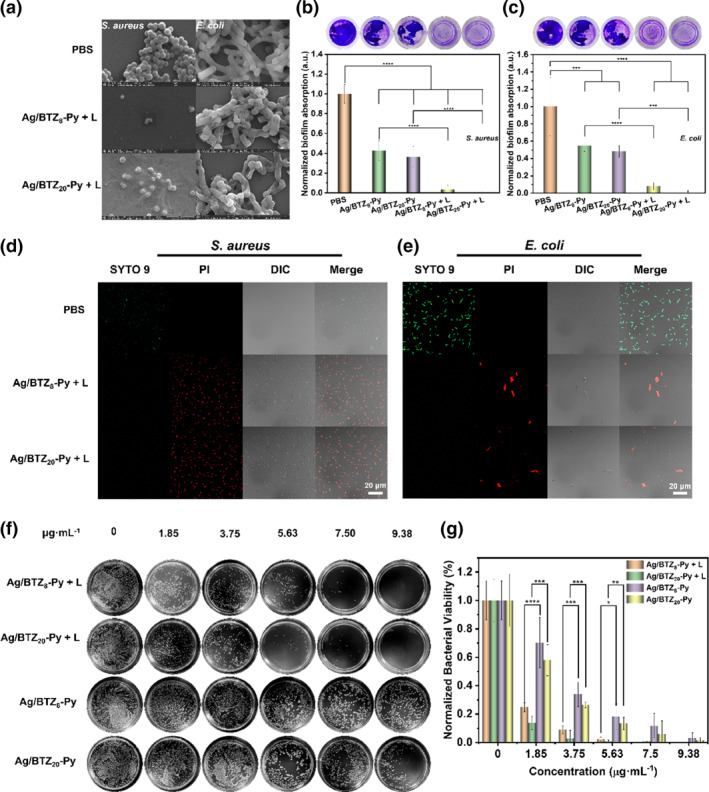
(a) Morphology of *S. aureus* and *E. coil* incubated with control PBS, Ag/BTZ_20_‐Py Light and Ag/BTZ_8_‐Py Light observed by SEM, respectively, (Light: irradiation at 515–530 nm light with 20 min). (b). Images of *S. aureus* and biofilms stained with Crystal Violet on culture dishes and Quantitative analysis of residual biofilm biomass. (c) Images of *E. coil* biofilms stained with Crystal Violet on culture dishes and Quantitative analysis of residual biofilm biomass. (d and e) Fluorescence images showing Ag/BTZ_8_‐Py and Ag/BTZ_20_‐Py antibacterial performance on *S. aureus* and *E. coil* with PI (red) to show dead bacteria and SYTO 9 (green) to indicate viable bacteria (scale bar: 20 μm). (f) Images of *S. aureus* colonies on LB agar plates after different treatments. (g) Antibacterial activity of Ag/BTZ_8_‐Py, Ag/BTZ_8_‐Py + L, Ag/BTZ_20_‐Py and Ag/BTZ_20_‐Py + L against *S. aureus*.

In response to the efficient generation of ^1^O_2_ and O_2_^−^, we further investigated the photodynamic antibacterial action of Ag/BTZ_n_‐Py. Firstly, we employed scanning electron microscopy (SEM) to examine the morphological changes of *Staphylococcus aureus* (*S. aureus*) and *Escherichia coli* (*E. coli*) after LED irradiation. SEM images revealed shrinkage, surface folding, and structural collapse on the surfaces of both bacteria, clearly demonstrating the damage and disruption of bacterial surface structures (Figure 3a), further confirming its antibacterial efficacy.

Compared to free‐floating bacteria, biofilms are more protected by extracellular polymeric substances, thus exhibiting higher resistance. We investigated the photodynamic eradication of biofilms. Initially, crystal violet staining was employed to quantify biofilm biomass. Both *S. aureus* and *E. coli* biofilms appeared deep blue. As shown in Figure 3b,c, Ag/BTZ_n_‐Py effectively removed the biofilms of *Escherichia coli* and *Staphylococcus aureus* under LED illumination.

To further quantify the residual biofilms, we utilized an enzyme + Linked immunosorbent assay (ELISA) to measure the optical density (OD) of crystal violet‐stained biofilm biomass. Ag/BTZ_n_‐Py exhibited a certain influence on the biofilm biomass of both *S. aureus* and *E. coli* under no light exposure. Under LED illumination, 30 μg/mL of Ag/BTZ_n_‐Py demonstrated highly efficient anti‐biofilm efficacy against *S. aureus* and *E. coli*. As depicted in Figure 3b, Ag/BTZ_8_‐Py reduced *S. aureus* biofilm biomass by 96%, while BTZ_20_‐Ag reduced it by 100%. Similarly, as shown in Figure 3c, Ag/BTZ_8_‐Py reduced *E. coli* biofilm biomass by 92%, while BTZ_20_‐Ag reduced it by 100%. Under LED illumination, Ag/BTZ_n_‐Py almost completely removed the biofilms of both *S. aureus* and *E. coli*.

Subsequently, we conducted imaging analysis of treated *S. aureus* and *E. coli* to understand the outstanding antibacterial performance of Ag/BTZ_n_‐Py. As illustrated in Figure 3d,e, live bacteria were labeled with green fluorescence using SYTO 9, while dead bacteria were labeled with red fluorescence using propidium iodide. Compared to the untreated control group, a significant increase in red fluorescence was observed when *S. aureus* and *E. coli* were treated and incubated with Ag/BTZ_n_‐Py. In contrast, no red fluorescence emission was observed in the blank control experiment, with bacteria exhibiting green fluorescence signals from SYTO 9. This indicates the excellent antibacterial properties of Ag/BTZ_n_‐Py.

We further investigated their antibacterial efficacy against Gram‐positive (G+) *Staphylococcus aureus* and Gram‐negative (G‐) *Escherichia coli*. Initially, we employed a plate counting method to detect the bactericidal efficacy of these compounds against G+ *Staphylococcus aureus* and G‐ *Escherichia coli*. As shown in Figure 3f and Figure S16a, all compounds demonstrated the ability to kill bacteria and inhibit high‐concentration proliferation under dark conditions. It is worth noting that the bactericidal efficacy of these compounds was significantly enhanced under light irradiation, with G+ bacteria being more susceptible than G‐bacteria. The difference in antibacterial efficacy can be attributed to the different membrane structures of G‐ and G+ bacteria. Interestingly, Ag/BTZ_20_‐Py exhibited more pronounced antibacterial activity than Ag/BTZ_8_‐Py.

To further validate this result and more accurately assess the antibacterial activity of all compounds, we further determined the minimum inhibitory concentration (MIC) against representative bacteria (*S. aureus* and *E. coli*). As illustrated in Figure 3g and Figure S16b, in the absence of light irradiation, the MIC of Ag/BTZ_n_‐Py against *S. aureus* was found to be 3.00 μg/mL (*n* = 8) and 2.43 μg/mL (*n* = 20). Correspondingly, for *E. coli*, the MIC was *ca.* 5.15 μg/mL (*n* = 8) and 4.25 μg/mL (*n* = 20), respectively. Upon exposure to light irradiation, the antibacterial activity of Ag/BTZ_n_‐Py was significantly enhanced. Specifically, the MIC of Ag/BTZ_8_‐Py was 0.93 μg/mL against *S. aureus* and 1.60 μg/mL against *E. coli*. Furthermore, the MIC of Ag/BTZ_20_‐Py was *ca.* 0.84 μg/mL against *S. aureus* and 1.45 μg/mL against *E. coli*. Notably, the MIC of Ag/BTZ_20_‐Py against *S. aureus* and *E. coli* were both lower than the those of Ag/BTZ_8_‐Py against *S. aureus* and *E. coli*. The enhanced antibacterial efficiency of Ag/BTZ_20_‐Py was attributed to the improved Ag^+^ release and augmented ROS generation upon light irradiation as mentioned above.

### 2.4 In vivo application of Ag/BTZ_n_‐Py (*n* = 8, 20)@Carb to treat a bacteria‐infected skin wound

**FIGURE 4:**
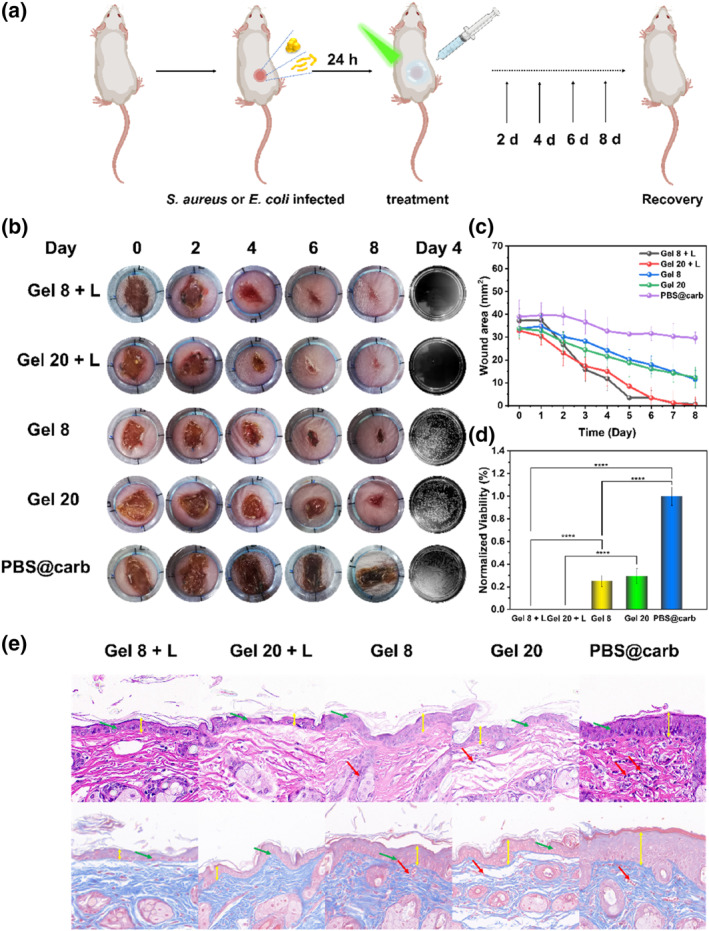
(a) Schematic Diagram of the Experimental Procedure for Mouse Wound Infection with *Staphylococcus aureus* and *Escherichia coli*. (b) Representative photographs of wounds infected by *S. aureus* biofilms and treated with PBS@carb + L, **Gel 8**, **Gel 20**, **Gel 8** + L and **Gel 20** + L under 515–530 nm LED for 8 days. (c) Infected area changes under different treatments. (d) Quantitative analysis of bacterial counts in wound tissues of day 4. The results are expressed as mean ± SD (*n* = 4, ****p < 0.0001 ***p < 0.001, **p < 0.01). (e) H&E and Masson‐stained wound slices on day 8. Histopathological evaluation of wound tissues treated with different groups (**Gel 8**, **Gel 20**, **Gel 8** + L, **Gel 20** + L) at day 8 (scale bar: 20 μm) (boundary of epithelium and dermis, yellow arrow; inflammatory cell, red arrow; hair follicles, green arrow).

As shown in Figure 4a, we selected a bacterial wound infection model using both *S. aureus* and *E. coli* to further evaluate the in vivo antibacterial activity of Ag/BTZ_n_‐Py. We created full‐thickness wounds with a diameter of approximately 8 mm on the backs of mice. To facilitate topical administration, we prepared Ag/BTZ_n_‐Py using Carbopol 941 as the matrix. This hydrogel exhibited a semi‐solid, transparent, and finely textured appearance (Figure S17).

Separate skin infection models were established for *S. aureus* and *E. coli* to assess the therapeutic effects. As depicted in Figure 4b and Figure S18a, the successful establishment of infected wounds was confirmed by the presence of yellow pus from *S. aureus* and white pus from *E. coli*.

Five groups of mice were treated with PBS@carb + Light(L), Ag/BTZ_8_‐Py@Carb (**Gel 8**) + L, Ag/BTZ_20_‐Py@Carb (**Gel 20**) + L, **Gel 8**, and **Gel 20**, covering the sites of *S. aureus* and *E. coli* infections. The effects of light exposure on bacterial infection were compared between the light and Dark groups. Wound dynamics were monitored every other day by recording the relative wound area to evaluate the therapeutic effects (Figure 4b and Figure S18a). The PBS@Carb control group exhibited a slower rate of wound healing, indicating the negative impact of bacterial infection on wound healing. Wounds treated with **Gel 8**, **Gel 20**, **Gel 8** + L and **Gel 20** + L almost completely healed, consistent with the results of in vitro antibacterial activity. Quantitative assessment of wound healing based on daily wound area measurements (Figure 4c and Figure S18b) showed some improvement in the **Gel 8** and **Gel 20** treatment groups, while the **Gel 8** + L and **Gel 20** + L groups achieved complete wound closure (100%).

On the fourth day, we used the plate counting method to determine the survival of *S. aureus* and *E. coli* at the infection sites (Figure 4d and Figure S18c). Compared to the other groups, bacterial counts were significantly reduced in the **Gel 8** + L and **Gel 20** + L groups.

On the eighth day, we examined the wounds infected with *S. aureus* after treatment with **Gel 8** + L and **Gel 20** + L. We used H&E staining and Masson staining (Figure 4e) to evaluate the formation and recovery of skin granulation tissue after treatment for *S. aureus* infection. Compared to the other groups, the mice treated with **Gel** 8 + L and **Gel 20** + L exhibited the highest levels of re‐epithelialization and dermal regeneration, minimal scarring, minimal infiltration of inflammatory cells, and significant improvements in hair follicle and blood vessel regeneration.

### 2.5 Biosafety evaluation of Ag/BTZ_n_‐Py (*n* = 8, 20)@Carb gel.

**FIGURE 5:**
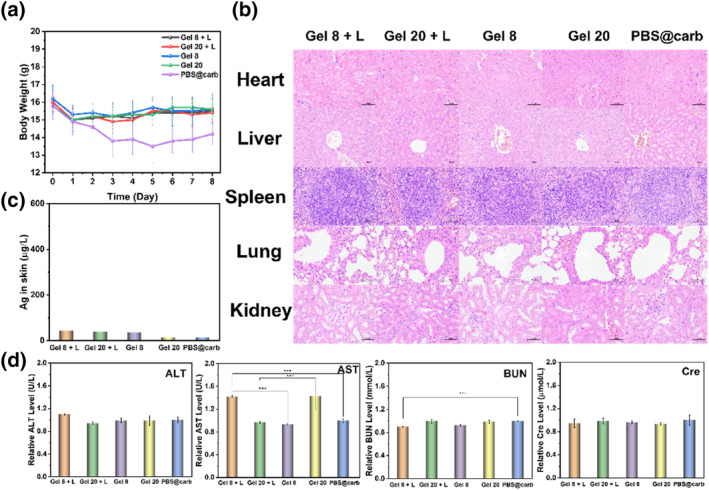
(a) Changes in mice body weight under different treatments. Data are presented as mean ± SD (*n* = 4). (b) H&E staining analysis of the back skin of mouse from different treatment groups. Scale bar: 50 μm. (c) The silver content in the skin wound after various treatments on day 8. (d) Hepatotoxicity and nephrotoxicity evaluation by measuring the serum levels of ALT, AST, BUN, and Cre after various treatments.

To comprehensively assess the translational potential of **Gel n** in the biomedical field, we further validated its biosafety. Throughout the treatment period, we closely monitored changes in mice body weight (Figure 5a and Figure S19a). No significant deviations were observed, indicating that the treatment had no long‐term adverse effects on the physiological status of the mice.

H&E staining was performed on local wound tissue and major organs (heart, liver, spleen, lung, kidney) of *S. aureus* on day 8. At the same time, H&E staining was performed on local wound tissue and major organs (heart, liver, spleen, lung, kidney, skin) of *E. coli* on day 10. No significant pathological changes were observed, confirming the non‐toxic effects of Ag/BTZ_n_‐Py (*n* = 8, 20) on the liver and kidneys (Figure 5b and Figure S19b), demonstrating good biosafety. Additionally, we used ICP‐OES technology to measure the silver content in skin (Figure 5c and Figure S19c). Encouragingly, no silver was detected in any samples, indicating rapid metabolism of **Gel n** in the mouse body and avoiding potential toxicity from silver accumulation.

To further assess the potential toxicity on major metabolic organs such as the liver and kidneys, we measured representative biomarkers associated with hepatotoxicity and nephrotoxicity. Gratifyingly, all indicators were within normal ranges (Figure 5d and Figure S19d), further confirming the biosafety of **Gel n**.

In conclusion, **Gel n** has demonstrated excellent biosafety, providing strong support for its clinical translation and potential application in treating infected wounds.

## 3 SUMMARY

In summary, we have fabricated a type‐I photosensitizer based metallo‐supramolecular nanofibers. The metallo‐supramolecular nanofibers were self‐assembled by type‐I photosensitizers with pyridine moieties BTZ_n_‐Py (*n* = 8, 20) and chemical antibacterial agents Ag^+^ driven by metal‐coordination interactions. Upon light irradiation, the prepared nanofibers could not only generate ^1^O_2_, but also type‐I ROS O_2_^•−^, which showed enhanced efficient antibacterial inhibition. Moreover, the acid‐ and photoactive Ag^+^ release endowed the nanofibers with controlled‐release property, which exhibited biosafety to the mammal cells. Moreover, it was found that the alkyl chain lengths in the type‐I photosensitizer affected the packing density of the nanofibers, which lead to different Ag^+^ releasing abilities. In in vivo and in vitro experiments, the biocompatible **Gel n** (*n* = 8, 20) gels were obtained by mixing Ag/BTZ_n_‐Py (*n* = 8, 20) nanofibers with Carbopol hydrogels. Due to the synergistic therapy between type‐I PDT and chemical antibacterial agents Ag^+^, the formed **Gel 20** exhibited outstanding antibacterial performance and excellent biosafety, which showed great promise in biomedical applications.

## CONFLICT OF INTEREST STATEMENT

There are no conflicts of interest.

## ETHICS STATEMENT

All animal experiments detailed in the manuscript were conducted in strict accordance with the guidelines approved by the Ethical Committee of Shandong University. The relevant production license number is SCXK 2019‐0001, and the use license number is SYXK 2019‐0005.

## Supporting information

Supporting Information S1

## Data Availability

The data that supports the findings of this study are available in the supplementary material of this article.
