# Supplementary material for: Metallo‐supramolecular nanofibers based on type‐I photosensitizer for synergistic antibacterial therapy
Source: Smart Mol. 2024 Dec 1;2(4):e20240037. doi: 10.1002/smo.20240037 (PMC12118169; doi:10.1002/smo.20240037)
Supplement: Supplementary file 1 — Supporting Information S1 [file SMO2-2-e20240037-s001.docx]

**Supplementary Material for**

**Metallo-Supramolecular Nanofibers Based on Type-I Photosensitizer for Synergistic Antibacterial Therapy**

Shibo Lyu^1,2^, Lukun Li^1^, Jingdong Gao^3^, Dapeng Liu^1^*, Fengling Song^1^*.

1. Institute of Frontier Chemistry, School of Chemistry and Chemical Engineering, Shandong University, Qingdao, 266237, P.R. China
2. Department of Chemistry, National University of Singapore, 117543, Singapore.
3. School of Life Sciences, Shandong University, Qingdao, Shandong, 266237, China.

# Materials and Instrumentation.

The chemicals 4,8-dibromo-6-(2-ethylhexyl)-[1,2,5]thiadiazole[3,4-F]benzotriazole and 4,8-dibromo-6-(2-octyldodecyl)-[1,2,5]thiadiazole[3,4-F]benzotriazole were purchased from Zhiyan Company. Other chemicals used include 4-pyridineboronic acid pinacol ester, Tetrakis(triphenylphosphine)palladium, silver trifluoromethanesulfonate, 1,3-diphenyl isobenzofuran (DPBF), and 2,2,6,6-tetramethylpiperidine (TEMP), 2-(3,6-Diamino-9H-xanthene-9-yl)-benzoic acid methyl ester (DHR 123), 2,2-dimethyl-3,4-dihydro-2H-pyrrole 1-oxide (DMPO).

Ultraviolet-visible (UV-vis) measurements were conducted using a HITACHI UH5300 spectrophotometer. Fluorescence spectra (FL) of the organic ligands BTZ_n_-Py (n = 8, 20) and Ag/BTZ_n_-Py (n=8, 20) in degassed THF solution (0.5 mg·mL^-1^) were obtained using a HITACHI F-4700 spectrofluorometer, with a Xe lamp and picosecond pulsed diode laser (500 nm) as the excitation source. The optical path length in the reported UV-vis and FL spectra was 1 cm unless otherwise specified. inductively coupled plasma-optical emission spectroscopy (ICP-OES) data were tested by Agilent 7700icpms instrument. X-ray Photoelectron Spectroscopy (XPS) was tested by the Thermo Fisher ESCALAB Xi+ instrument. Fourier-transform infrared spectroscopy (FT-IR) was performed on a Nicolet 6700 FT-IR spectrometer. The surface area was determined by nitrogen adsorption isotherm and BET method on Micromeritics ASAP 2460. The cyclic voltammetry experiment was conducted using a three-electrode system. A glassy carbon electrode served as the working electrode, a platinum wire electrode as the auxiliary electrode, and an Ag/Ag⁺ electrode as the reference electrode. The measurements were carried out in THF containing 0.1 M (n-Bu)₄N⁺PF₆⁻ as the supporting electrolyte. Fc/Fc⁺ was used as the external reference.

Multinuclear NMR spectra, including ^1^H NMR, were recorded on a Bruker Ascend-400 spectrometer. High-resolution ESI-TOF-MS measurements were conducted on an LTQ-Orbitrap mass spectrometer with ETD. Electron spin resonance (ESR) measurements were performed at room temperature (25 °C) using an EMXnano spectrometer with a 1 G field modulation, 100 G scan range, and 20 mW microwave power. TEMP was used as a spin trap for ^1^O_2_. DMPO was used as a spin trap for O_2_^.-^. Transmission electron microscopy (TEM) images were captured with an FEI Tecnai G2 F20 operating at 200 kV acceleration voltage. Fluorescence images of bacteria were taken using a Zeiss LSM 900 confocal fluorescence microscope.

# Synthesis of the Organic Ligand BTZ_20_-Py.

**
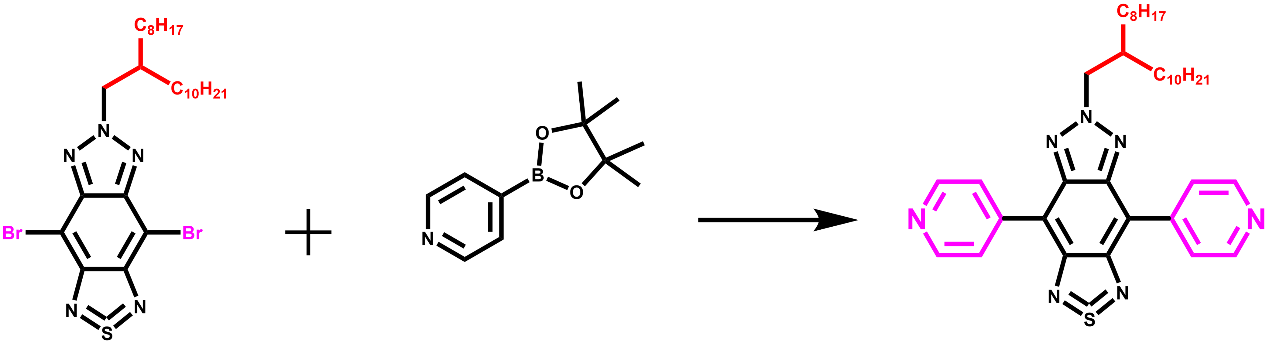
**

**Scheme S1.** Synthetic route of the BTZ_20_-Py.

The ligand BTZ_20_-Py was synthesized via a Suzuki-coupling reaction. To begin, 4,8-dibromo-6-(2-ethylhexyl)-[1,2,5]thiadiazole[3,4-F]benzotriazole (100 mg, 0.217 mmol) was dissolved in degassed toluene (40 mL) and H₂O (10 mL). Then, 4-(4,4,5,5-tetramethyl-1,3,2-dioxaborolan-2-yl)pyridine (97.79 mg, 0.477 mmol) along with the catalyst Pd[P(C_6_H_5_)_3_]_4_ and K₂CO₃ were added at room temperature. The mixture was heated to 70 °C and stirred for 24 hours. After the reaction, the mixture was cooled to room temperature and filtered through a short silica gel column to remove precipitates. The filtrate was concentrated under vacuum and purified on a silica gel column using an ethanol/CH₂Cl₂ (1:10) eluent, yielding 44 mg of BTZ_20_-Py. Yield: 48%. ^1^H NMR (400 MHz, CDCl₃): δ 8.88 (d, 2H), 8.47 (d, 2H), 4.8(d, 2H), 2.5(s, 1H), 1.69-1.60 (m, 4H), 0.092(t, 6H), 0.88-0.83(m, 6H). ESI-MS (m/z): calcd. for C36H49N7S [M+H]^+^, Calcd, 612.38; found, 612.38.

# Fabrication of the Supramolecular nanofiber Ag/BTZ_n_-Py (n = 8, 20).

The organic ligands BTZ_n_-Py (28 mg, 0.046 mmol) and Ag(OTf) (11.76 mg, 0.046 mmol) were accurately weighed and stirred in THF at room temperature for 2 hours, avoiding light. After the reaction, a red precipitate formed, which was then centrifuged with THF until the supernatant was colorless. Vacuum-dry the sample and collect the resulting red solid powder BTZ_n_-Py.

# Bacterial culture

Transfer a single bacterial colony from Luria-Bertani (LB) agar medium to 4 mL of LB broth. Incubate the bacterial culture at 37°C with shaking at 200 rpm for 24 hours to reach the logarithmic growth phase. In this experiment, Staphylococcus aureus (*S. aureus*, ATCC2923) and Escherichia coli (*E. coli*, ATCC25922) were used as representatives of Gram-positive and Gram-negative bacteria, respectively. After incubation, the bacteria were harvested by centrifugation at 6000 rpm for 5 minutes, washed three times with PBS, and re-hung in PBS. The absorbance at 600 nm was measured with an ultraviolet-visible spectrophotometer and the bacterial suspension was diluted to a final concentration of 1×10^9^ CFU/mL.

# Bacterial staining

Add 800 μL of a PBS solution containing Ag/BTZ_n_-Py at a concentration of 0.5 mg·mL^-1^ to a bacterial suspension (1000 μL) with a density of 1×10^9^ CFU/mL. Incubate the mixture in a constant temperature oscillating incubator at 37°C and 200 rpm for 30 minutes. Then, expose the bacterial suspensions containing Ag/BTZ_n_-Py to a 510-530 nm LED light (20 mW·cm^-2^) for 20 minutes. After treatment, centrifuge the bacterial suspensions at 6000 rpm for 5 minutes and resuspend them in normal saline. Following the manufacturer's instructions, add SYTO 9 and PI (Live/Dead Bacterial Viability Kit) and stain the samples in the dark with shaking for 30 minutes. After staining, wash the samples (10 μL) three times with PBS, place them on a slide, and observe under a Zeiss LSM 900 laser confocal microscope. The excitation wavelength for SYTO 9 is 480 nm with an emission range of 510-540 nm, and for PI, the excitation wavelength is 490 nm with an emission range of 620-650 nm.

# Bacterial inhibition was assessed by LB plate dilution

Dilute the bacterial suspension in PBS to a concentration of 1×10^7^ CFU/mL. Then, mix the diluted suspension with Ag/BTZ_n_-Py solutions at varying concentrations. Stir the mixture at 200 rpm and incubate at 37°C for 30 minutes to allow the photosensitizer to fully adhere to the bacterial surface. After incubation, expose the samples to 515-530 nm LED light (20 mW·cm^-2^) for 20 minutes. Subsequently, further dilute the bacterial suspension to 1×10^4^ CFU/mL. Take 50 μL of the diluted suspension and spread it evenly on LB agar plates using a spreader. Incubate the plates statically at 37 °C for 24 hours, then count the colonies.

# Inhibitory activity on Mature Biofilms

Dilute the bacterial suspension in PBS to a concentration of 1×10^7^ CFU/mL. Then, add equal concentrations of Staphylococcus aureus and Escherichia coli to 96-well plates containing LB medium with 1 % glucose (200 μL per well). Incubate at 37°C for 72 hours to allow biofilm formation. After biofilm development, add different treatments to the wells: Ag/BTZ_n_-Py (dark) and Ag/BTZ_n_-Py (light), and incubate for an additional 12 hours.

After incubation, remove the supernatant and wash the biofilms 2-3 times with PBS to remove any remaining medium. Then, use the crystal violet assay to determine the remaining bacterial biomass within the biofilms. Add 100 μL of methanol to each well and incubate for 15 minutes. Remove the methanol and let the well plate air dry. Next, add 100 μL of 1 % crystal violet solution to each well and stain at room temperature for 15 minutes. After staining, wash with PBS to remove excess crystal violet, eliminating non-specific staining. Following the removal of excess crystal violet, wash the biofilms again with PBS and dry at 37 °C. To dissolve the crystal violet, add 100 μL of 33 % glacial acetic acid solution to each well and incubate at 37°C for 30 minutes. The absorbance of the crystal violet at 590 nm was then measured using an enzyme-labeled instrument.

# Preparation of hydrogels

To prepare a suitable hydrogel, Carbopol 941 was chosen as the base material. First, weigh 1 g of Carbopol 941 powder and place it in a 50 mL beaker. Then, add 25 mL of deionized water under stirring conditions and allow it to swell overnight.

After swelling overnight, adjust the pH to approximately 7.4 by adding sodium bicarbonate (NaHCO₃) dropwise while stirring thoroughly to prepare a 2% Carbopol 941 hydrogel. Next, add PBS and Ag/BTZ_n_-Py aqueous solutions to the prepared Carbopol 941 gel, adjusting their concentrations to 0.5 mg/mL. Gently stir the mixtures for 10 minutes to ensure even distribution, forming Ag/BTZ_n_-Py (n = 8, 20)@Carb and PBS@Carb hydrogels.

# Animal model

All animal experiments are performed following the protocols approved by the Ethical Committee of Shandong University under production license number SCXK 2019- 0001 and use license number SYXK 2019-0005. Forty male Balb/c mice, aged 6-8 weeks and weighing 15-18 g, were randomly divided into eight groups of four. Four groups were used for Staphylococcus aureus (*S. aureus*) infections, and four groups were used for Escherichia coli (*E. coli*) infections. To induce wound infections, the mice were anesthetized, and circular wounds with a radius of 4 mm were created on their backs. The following day, 100 μL of S. aureus and E. coli (10^7^ CFU/mL) were injected into the wound sites, respectively, and the mice were allowed to incubate for 1-2 days. Subsequently, Ag/BTZ_n_-Py@Carb hydrogels were evenly applied to the infected wound areas, with separate groups treated with light exposure and without light exposure. The control groups had PBS@Carb applied to the wounds. The light-exposed groups were subjected to illumination from a 515-530 nm LED lamp (20 mW/cm^2^) for 15 minutes every other day, for a total of three exposures.

All animals were euthanized on the 8th or 10th day post-treatment. Tissues, including the heart, liver, spleen, lungs, kidneys, and wound tissues, were collected for H&E and Masson staining to assess the biosafety and toxicity of Ag/BTZ_n_-Py@Carb.

# Statistical analysis

The data were presented as mean ± s.d. (standard deviation) and obtained from a minimum of Four independent experiments. Statistical analysis was conducted using the one-way ANOVA test. p-value less than 0.05 was considered as statistically significant.

**
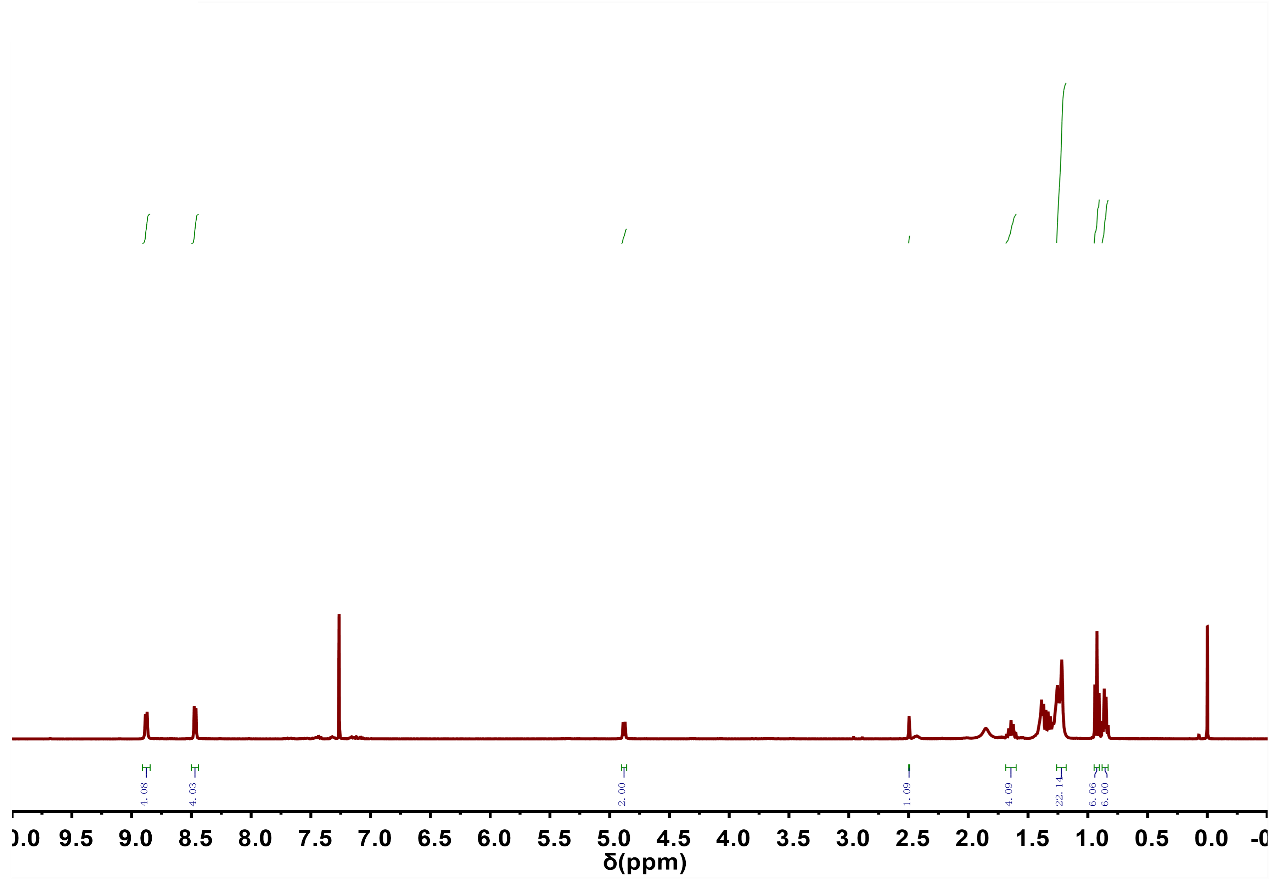
**

**Figure S1.** ^1^H NMR spectrum of BTZ_20_-Py in CDCl3 at 298 K.

**
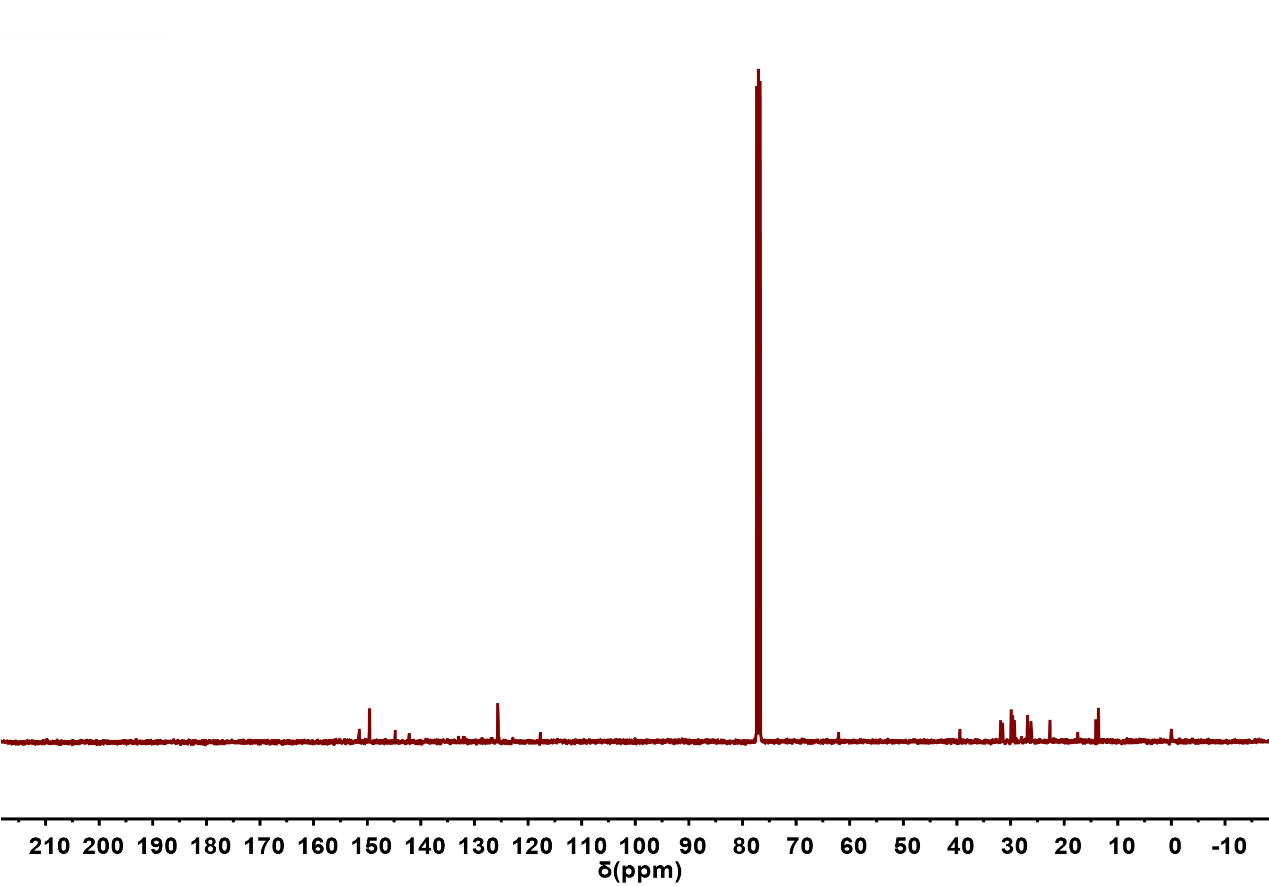
Figure S2.** ^13^C NMR spectrum of BTZ_20_-Py in CDCl3 at 298 K.

**
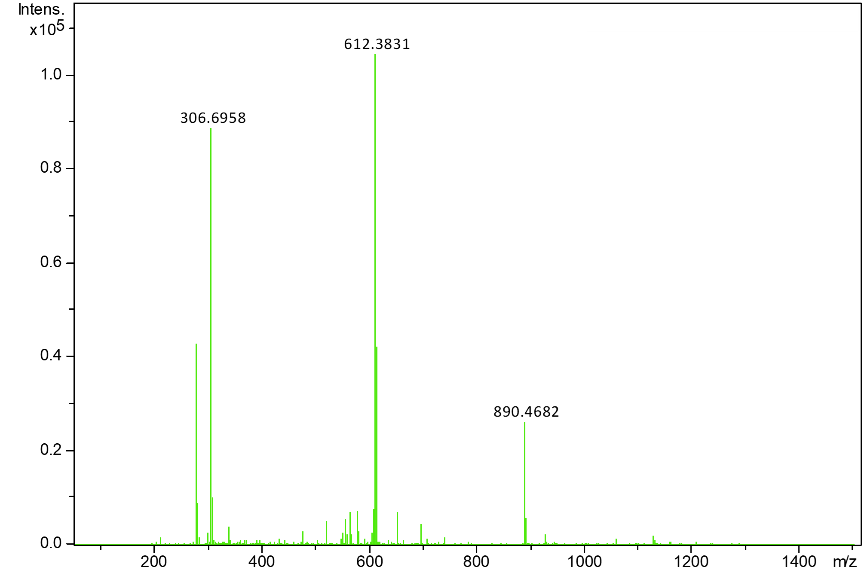
**

**Figure S3.** ESI-MS of BTZ_20_-Py.


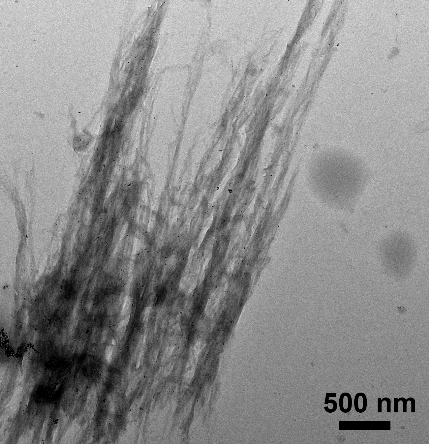


**Figure S4.** TEM images of Ag/BTZ_8_-Py.


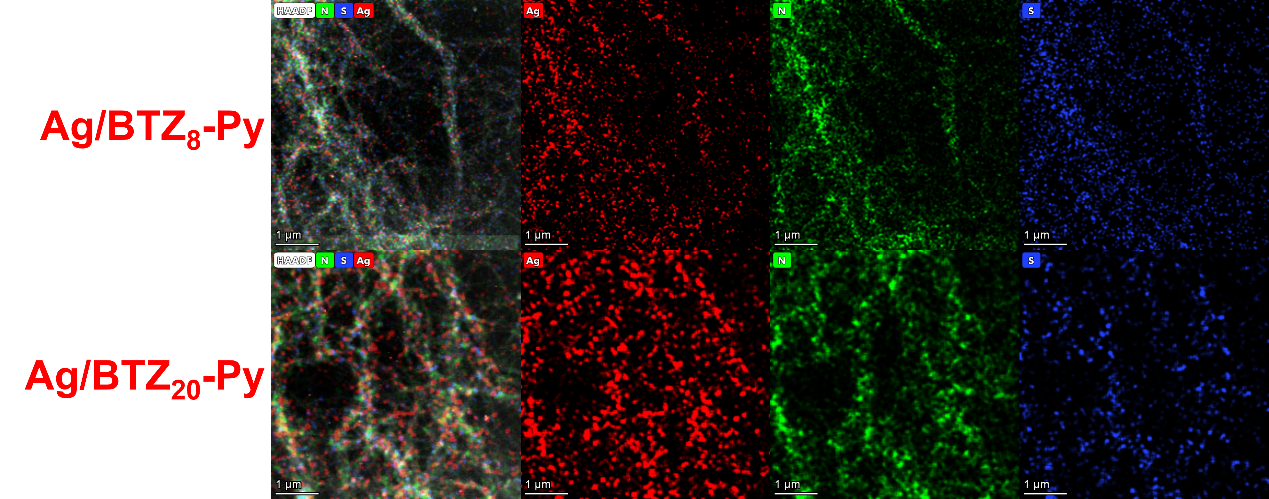


**Figure S5.** TEM–EDS mapping image (inset scale bar: 1 μm) of Ag/BTZ_8_-Py and Ag/BTZ_20_-Py.


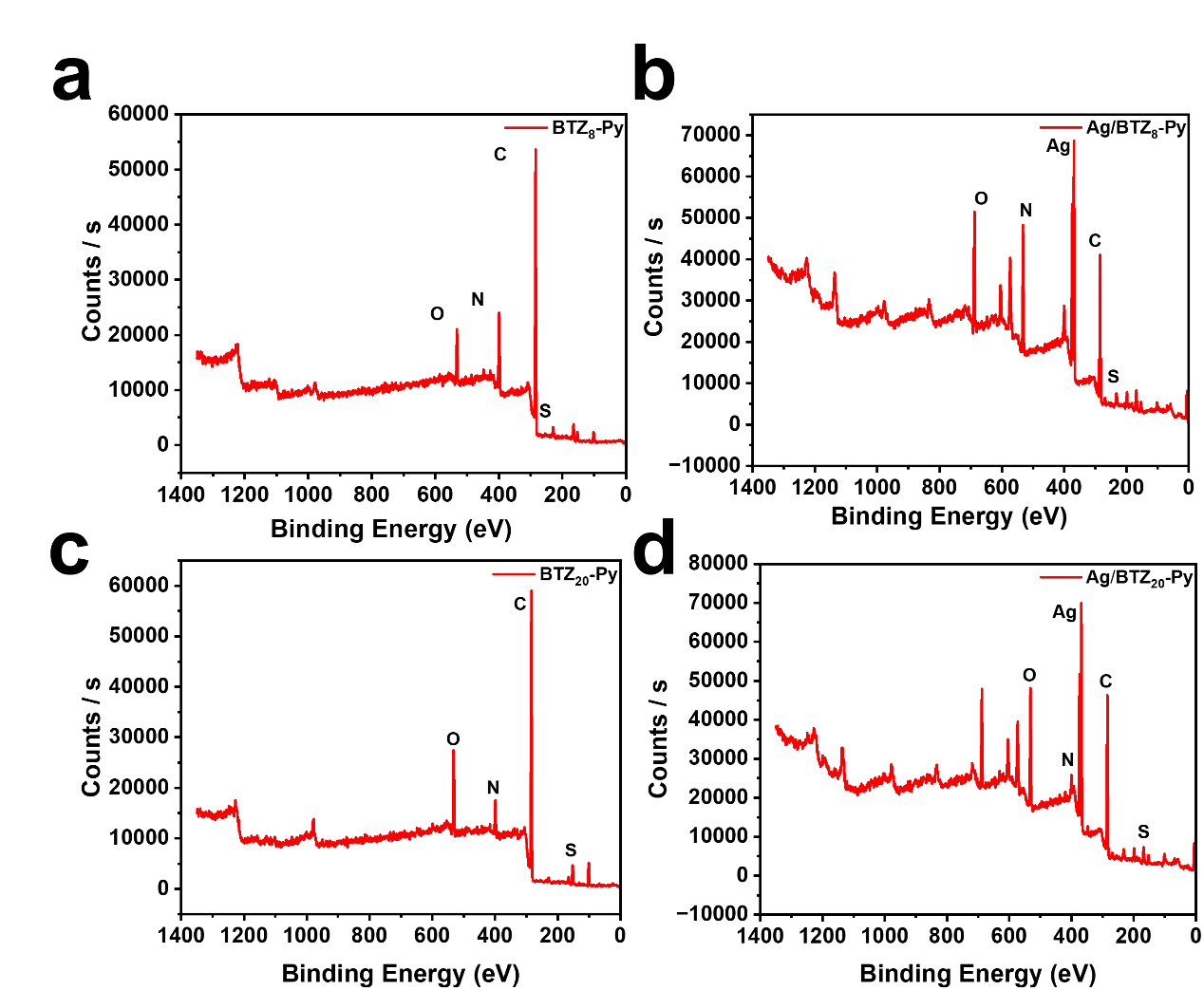


**Figure S6.** XPS survey spectra of BTZ_8_-Py (figure a), Ag/BTZ_8_-Py (figure b) and BTZ_20_-Py (figure c), Ag/BTZ_20_-Py (figure d).


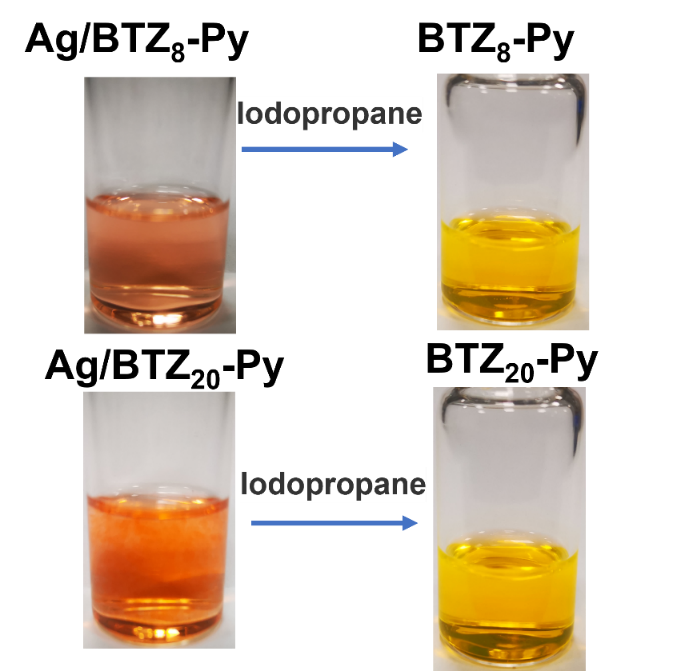


**Figure S7.** Dissociated BTZ_8_-Py and BTZ_20_-Py images in THF solution.


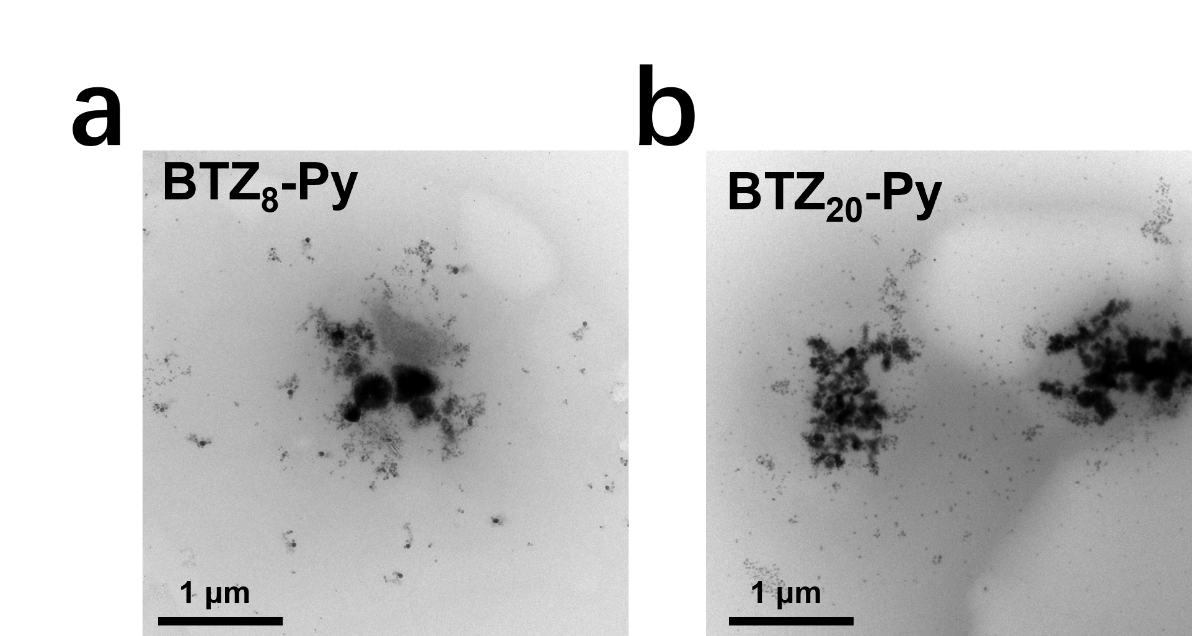


**Figure S8.** TEM image of BTZ_8_-Py (Figure a) and BTZ_20_-Py (Figure b) after Ag/BTZ_8_-Py and Ag/BTZ_20_-Py dissociation.


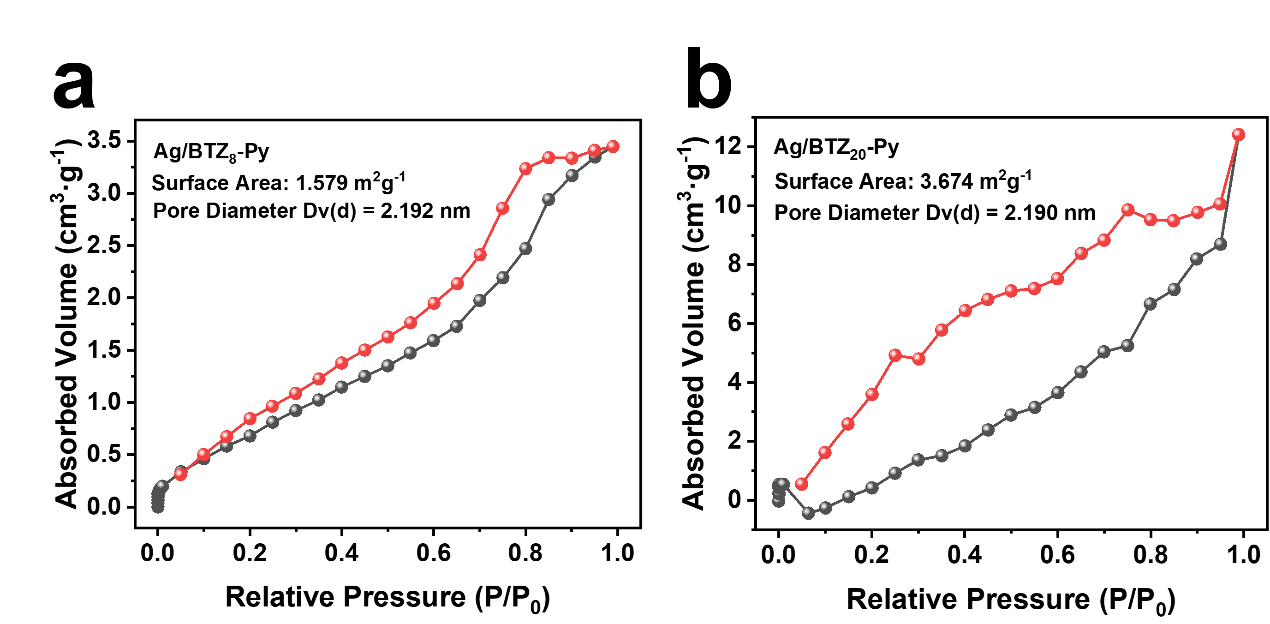


**Figure S9.** N_2_ adsorption–desorption isotherms of Ag/BTZ_8_-Py (Figure a) and Ag/BTZ_20_-Ag (Figure b).


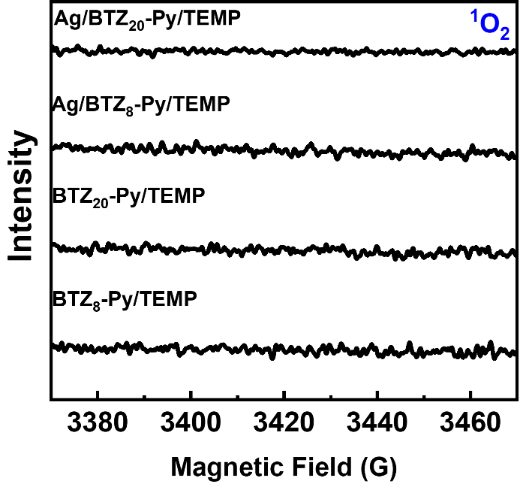


**Figure S10.** ESR spectrum of BTZ_8_-Py, BTZ_20_-Py, Ag/BTZ_8_-Py and Ag/BTZ_20_-Py using TEMP as an ^1^O_2_ trap in dark.


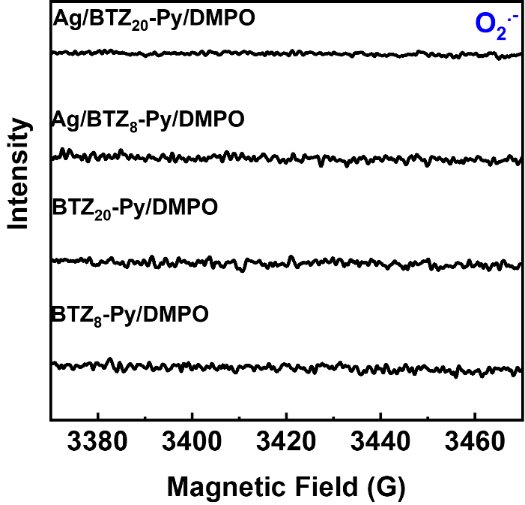


**Figure S11.** ESR spectrum of BTZ_8_-Py, BTZ_20_-Py, Ag/BTZ_8_-Py and Ag/BTZ_20_-Py using DMPO as an O_2_^.-^ trap in dark.


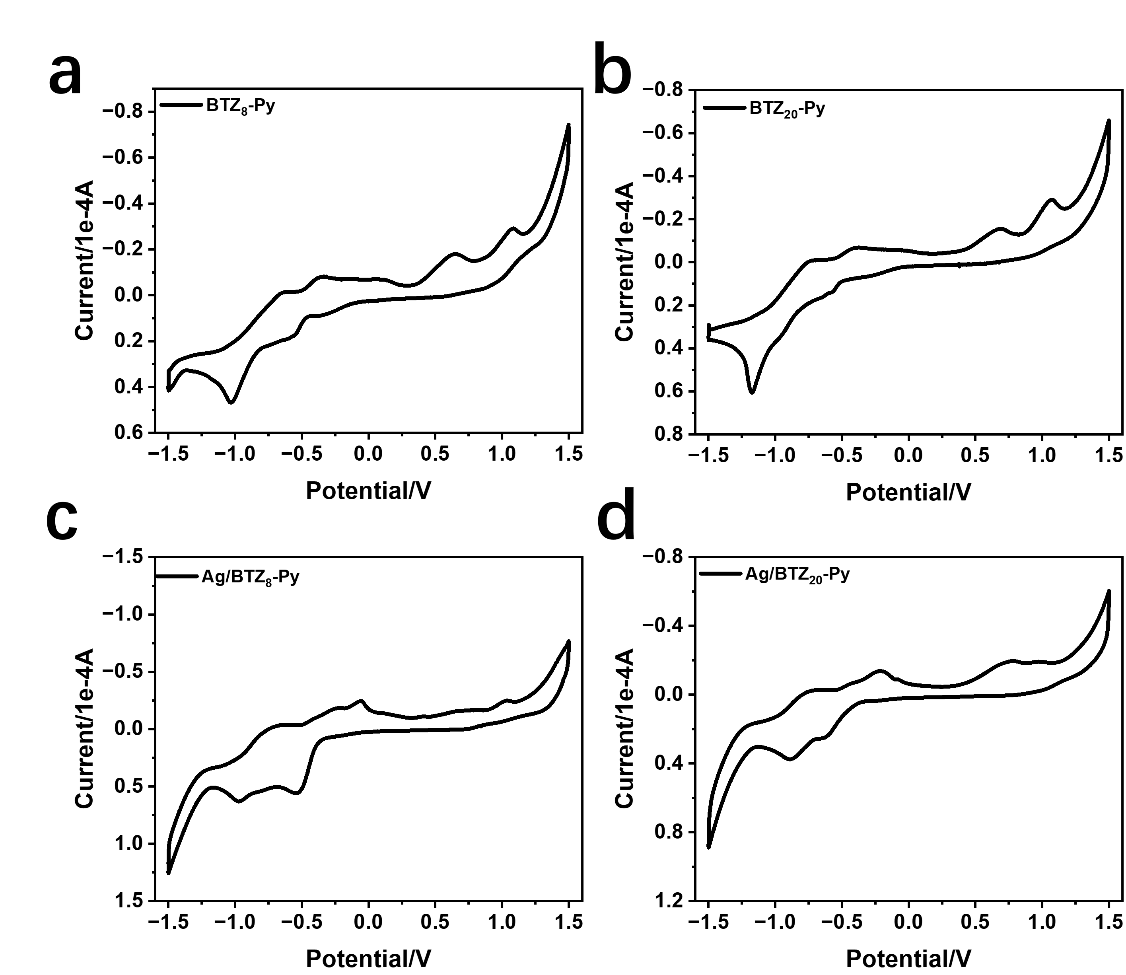


**Figure S12.** The cyclic voltammograms of BTZ_8_-Py (Figure a), BTZ_20_-Py (Figure b), Ag/BTZ_8_-Py (Figure c), and Ag/BTZ_20_-Py (Figure d) were recorded in THF with 0.1 M (n-Bu)_4_N^+^PF_6_^-^ as the supporting electrolyte, using Ag/Ag^+^ as the reference electrode, a glassy carbon as the working electrode, and platinum electrodes as the counter electrodes; Fc/Fc^+^ was used as an external reference.

**
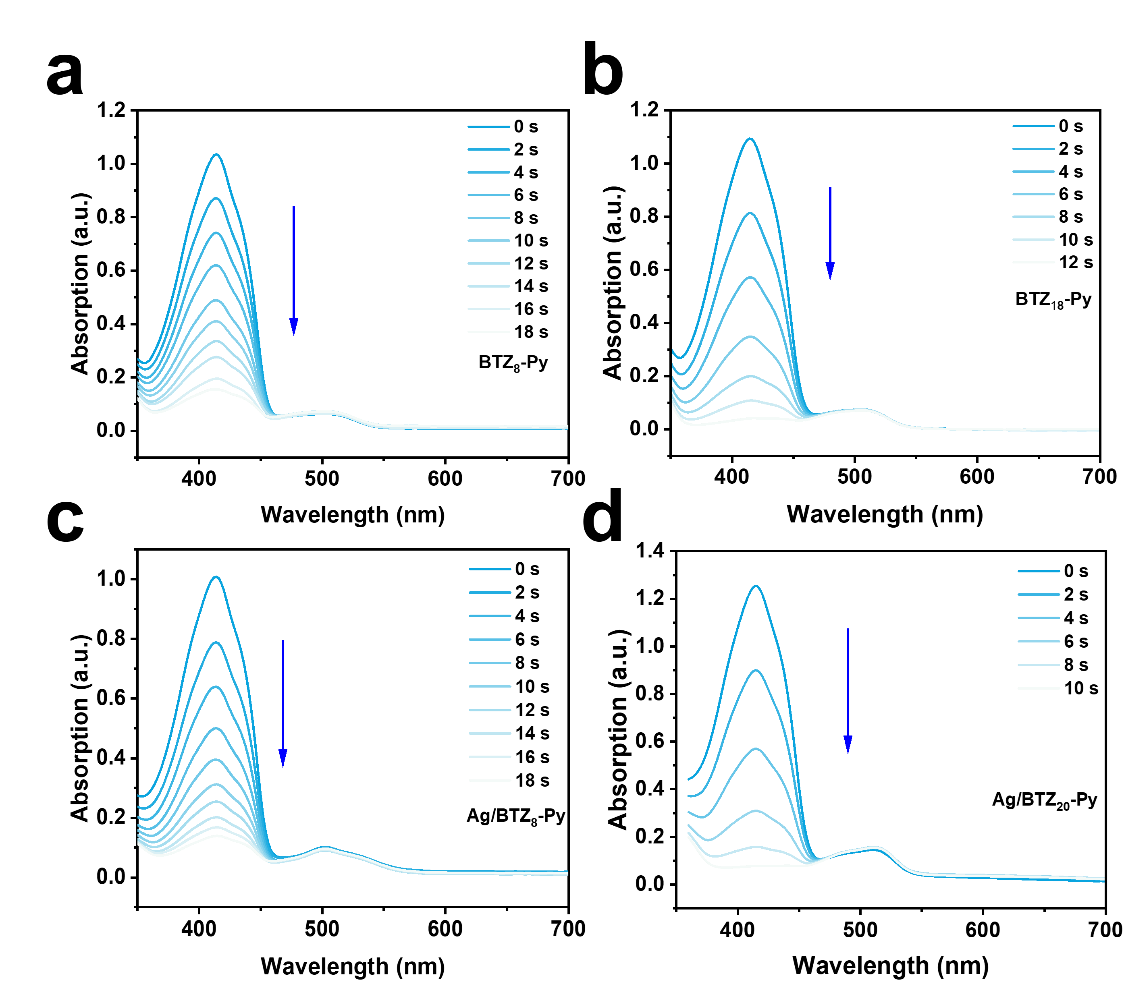
**

**Figure S13.** Absorption spectra of various times irradiation (0- 20 s) of BTZ_8_-Py (Figure a), BTZ_20_-Py (Figure b), Ag/BTZ_8_-Py（Figure c）and Ag/BTZ_20_-Py (Figure d) mixed with DPBF in THF solution.

**
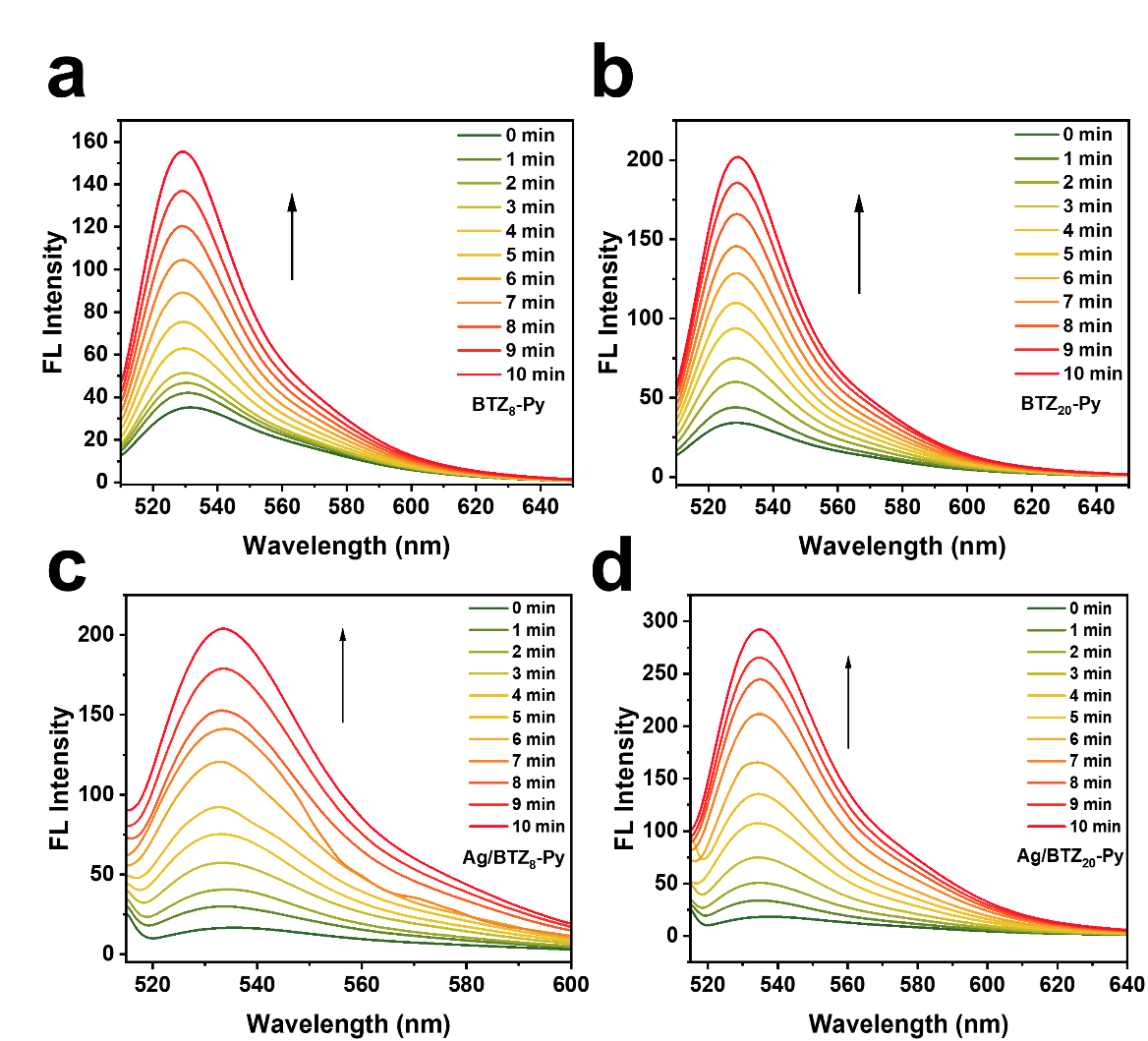
**

**Figure S14.** Fluorescence emission spectra of various times irradiation (0- 10 min) of BTZ_8_-Py (Figure a), BTZ_20_-Py (Figure b), Ag/BTZ_8_-Py (Figure c) and Ag/BTZ_20_-Py (Figure d) mixed with DHR 123.


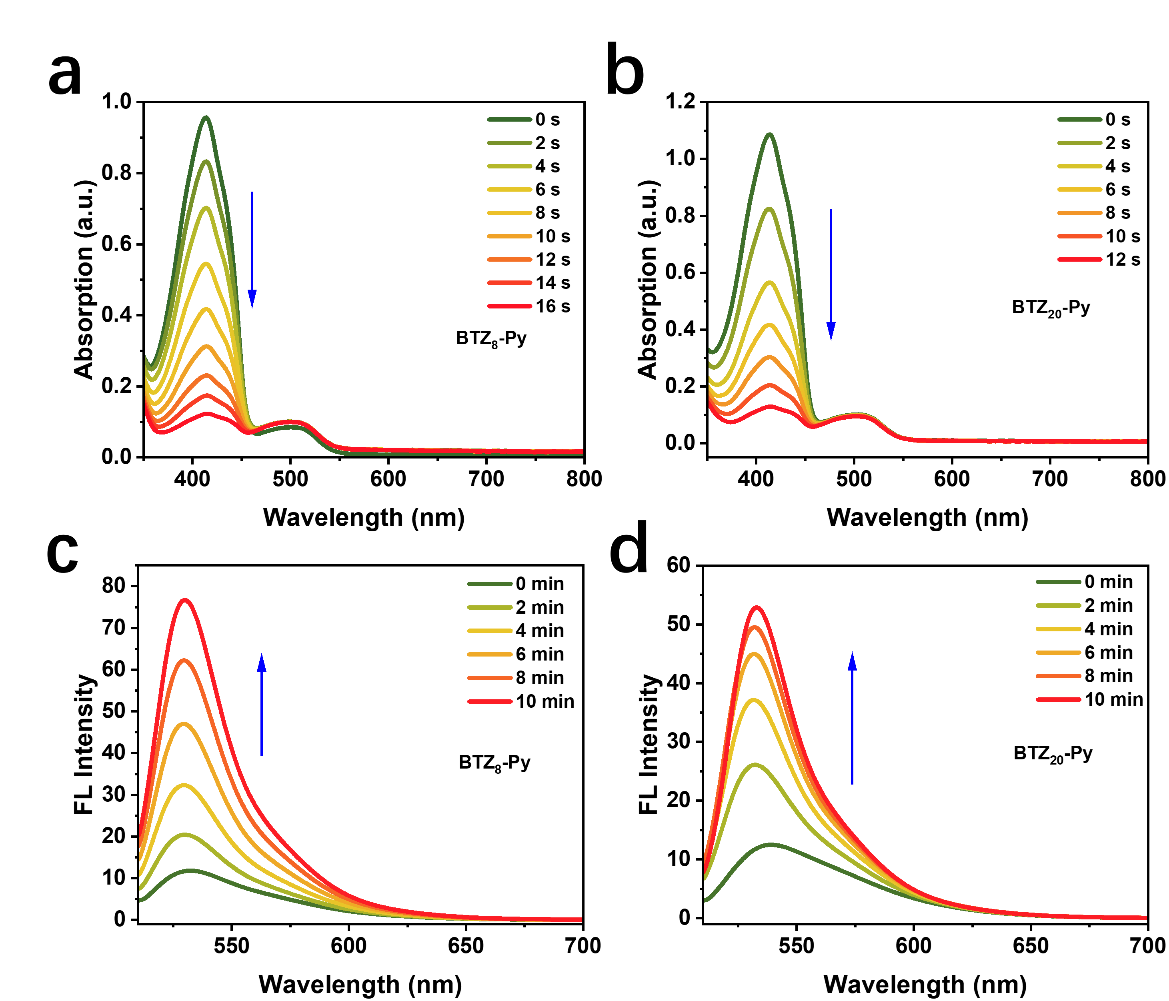


**Figure S15.** Absorption spectra of various times irradiation of BTZ_8_-Py （Figure a） and BTZ_20_-Py (Figure b) mixed with DPBF in THF solution after dissociation. Fluorescence emission spectra of various times irradiation (0- 10 min) of BTZ_8_-Py (Figure c), BTZ_20_-Py (Figure d) mixed with DHR 123 after dissociation.


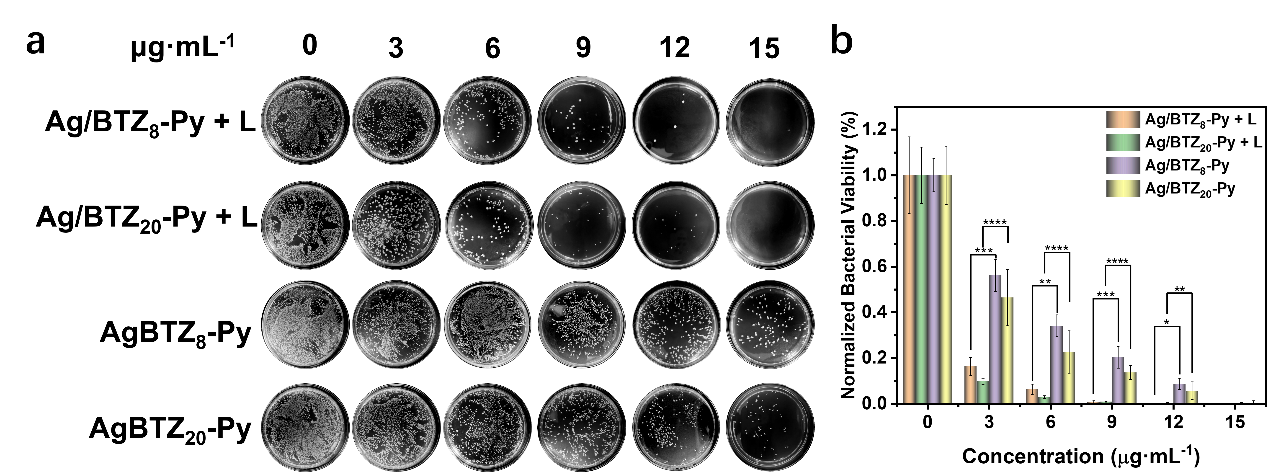


**Figure S16.** a) Images of *E. coil* colonies on LB agar plates after different treatments. b) Antibacterial activity of Ag/BTZ_8_-Py, Ag/BTZ_8_-Py + L, Ag/BTZ_20_-Py and Ag/BTZ_20_-Py + L against *E. coil*.


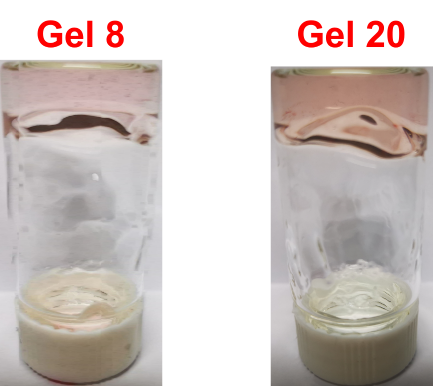


**Figure S17.** Photographs of **Gel 8** and **Gel 20**.


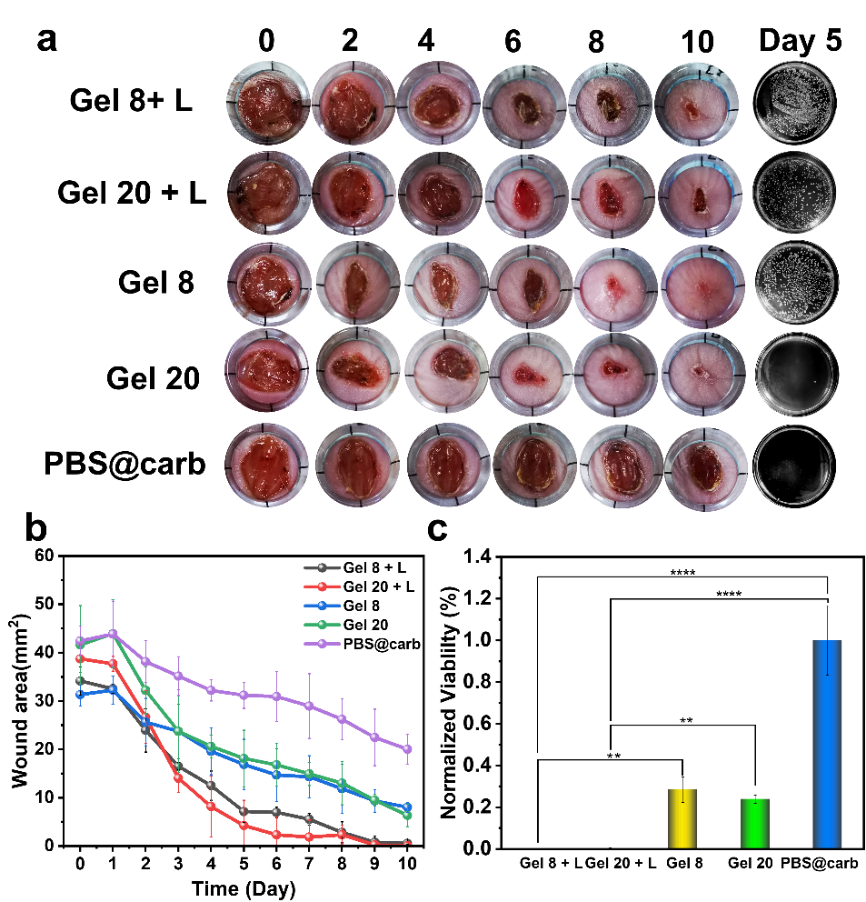


**Figure S18.** a) Images showing the wounds in an *E. coil* -infected mouse model after various treatments at different time points. b) Relative wound area of mice. c) Quantitative analysis of bacterial counts in wound tissues of day 4. The results are expressed as mean ± SD (n = 4, ****p < 0.0001 ***p < 0.001, **p < 0.01).


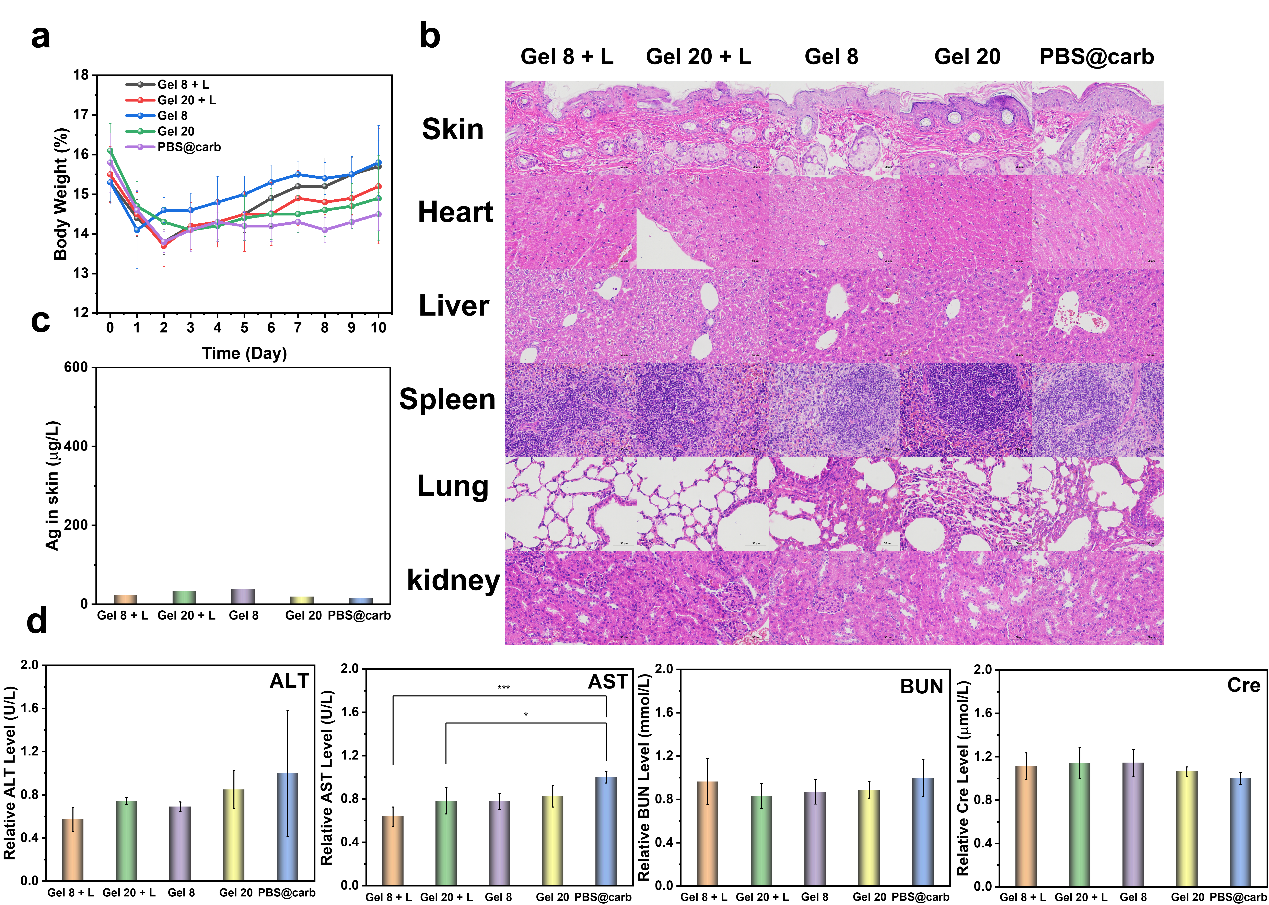


**Figure S19.** a) Changes in mice body weight under different treatments. Data are presented as mean ± SD (n = 4). b) H&E staining analysis of the back skin of mouse from different treatment groups. Scale bar: 50 µm. c) The silver content in the skin wound after various treatments on day 10. d) Hepatotoxicity and nephrotoxicity evaluation by measuring the serum levels of ALT, AST, BUN, and Cre after various treatments.
